# The missense mutation in Abcg5 gene in spontaneously hypertensive rats (SHR) segregates with phytosterolemia but not hypertension

**DOI:** 10.1186/1471-2156-6-40

**Published:** 2005-07-18

**Authors:** Jianliang Chen, Ashok Batta, Shuqin Zheng, Wayne R Fitzgibbon, Michael E Ullian, Hongwei Yu, Patrick Tso, Gerald Salen, Shailendra B Patel

**Affiliations:** 1 Division of Endocrinology, Diabetes and Medical Genetics, Medical University of South Carolina, STR 541, 114 Doughty Street, Charleston, SC 29403, USA; 2 Research Service and Medical Service, Department of Veterans Affairs Medical Center, East Orange, NJ 07019, USA; 3 Department of Pathology and laboratory Medicine, University of Cincinnati College of Medicine, Cincinnati, Ohio 45267. USA; 4 Division of Nephrology, Medical University of South Carolina, Charleston, SC 29403, USA

## Abstract

**Background:**

Sitosterolemia is a recessively inherited disorder in humans that is associated with premature atherosclerotic disease. Mutations in ABCG5 or ABCG8, comprising the sitosterolemia locus, *STSL*, are now known to cause this disease. Three in-bred strains of rats, WKY, SHR and SHRSP, are known to be sitosterolemic, hypertensive and they carry a missense 'mutation' in a conserved residue of Abcg5, Gly583Cys. Since these rat strains are also know to carry mutations at other genetic loci and the extent of phytosterolemia is only moderate, it is important to verify that the mutations in Abcg5 are causative for phytosterolemia and whether they contribute to hypertension.

**Methods:**

To investigate whether the missense change in Abcg5 is responsible for the sitosterolemia we performed a segregation analysis in 103 F2 rats from a SHR × SD cross. Additionally, we measured tail-cuff blood pressure and measured intestinal lipid transport to identify possible mechanisms whereby this mutation causes sitosterolemia.

**Results:**

Segregation analysis showed that the inheritance of the Gly583Cys mutation Abcg5 segregated with elevated plant sterols and this pattern was recessive, proving that this genetic change is responsible for the sitosterolemia in these rat strains. Tail-cuff monitoring of blood pressure in conscious animals showed no significant differences between wild-type, heterozygous and homozygous mutant F2 rats, suggesting that this alteration may not be a significant determinant of hypertension in these rats on a chow diet.

**Conclusion:**

This study shows that the previously identified Gly583Cys change in Abcg5 in three hypertension-susceptible rats is responsible for the sitosterolemia, but may not be a major determinant of blood pressure in these rats.

## Background

Sitosterolemia is an autosomal recessive disease, characterized by significantly increased plasma levels of plant sterols (such as sitosterol, campesterol), and is associated with premature atherosclerotic disease [1]. This disease has been mapped to a single locus, *STSL*, on human chromosome 2p21 [2,3]. Mutations in both alleles of one of two genes, ABCG5 or ABCG8, that comprise this locus, are now known to cause this disease [4-6]. No phytosterolemic patient with a single mutant ABCG5 allele and a mutant ABCG8 allele has been reported, suggesting these genes are not only linked physically, but their protein products may act as obligate heterodimers. ABCG5 and ABCG8 encode for sterolin-1 and sterolin-2 respectively. These genes are expressed in the liver, gall bladder and intestine and are implicated in determining biliary sterol excretion and selectivity of sterol absorption at the apical surfaces of the enterocytes [7-12]. Studies in mice deficient for Abcg5 or Abcg8, as well as one that over-expresses these genes, have confirmed that sterolins may be the major determinants of biliary sterol secretion [13-18]. Their role in preventing non-cholesterol sterol absorption in the intestine remains to be clarified.

Prior to the identification of the gene defects in sitosterolemia, the presence of sitosterolemia had been reported in the spontaneously hypertensive rat (SHR), although the molecular basis of this phenotype had not been investigated [19-25]. Sitosterolemia, hemolysis (a feature also present in human sitosterolemia) and an increased mortality when fed certain plant oils (enriched in plant sterols) have also been reported in SHR and SHRSP rats [20]. The SHR, SHRSP and their 'parental' strain, WKY rats have now been shown to carry a homozygous G1757T alteration in Abcg5 that results in a Gly583Cys change [26,27]. All of these rat strains are also sitosterolemic. However, all of these rats are highly in-bred and have been maintained by brother-sister matings [28]. These rats are likely to carry a number of mutated genes as part of their genetic burden. At least 4 'mutated' loci are known to be part of this burden; Cd36 [29], Srebp-1 [30], Kat-2 [31] and Abcg5 [26,27]. Although the Gly583Cys affects a highly conserved residue (conserved from Fugu to Man) [27], given the known genetic burden in the SHR line, it remains possible that genetic alterations at other loci may be responsible for the sitosterolemia. Support for this possibility has recently come from a quantitative trait genetic mapping study for sitosterolemia involving two mouse strains, C57Bl/6J and CASA/Rk [32,33]. Although the plasma sitosterol level was genetically determined in these mouse strains, loci that accounted for this did not map to the murine *STSL *and thus other unidentified genes were hypothesized to be involved [32,33].

We report here genetic and biochemical analyses of F2 rats from a SHR × SD cross to test the hypothesis that the Gly583Cys is responsible for the sitosterolemia. In addition, since hypertension is a known phenotype of the SHR and since feeding diets rich in plant sterols seem to decrease their life-span, we also investigated the possibility that this genetic change may also be important in playing a role in hypertension.

## Results

### Segregation analyses of plasma cholesterol and plant sterols

**Figure 1:**
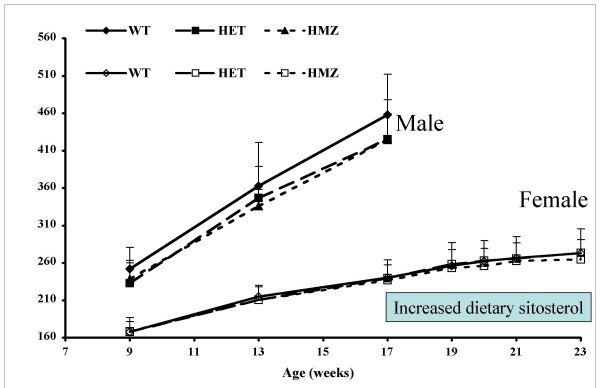
**Growth Curves of F2 mice. **The body weights of male (top set of lines) and female (bottom set of lines) F2 rats were monitored with time. At 17 weeks, most of the male F2 animals were sacrificed, or were used to determine intestinal absorption (see Fig. 5). At 17 weeks, female F2 rats were placed on a more defined plant sterol diet and their drinking water substituted with 1% NaCl. No differences in body weight gains between any of the Abcg5 genotypes (wild-type, WT; heterozygous, HET and homozygous mutant, HMZ) were observed.

**Figure 2:**
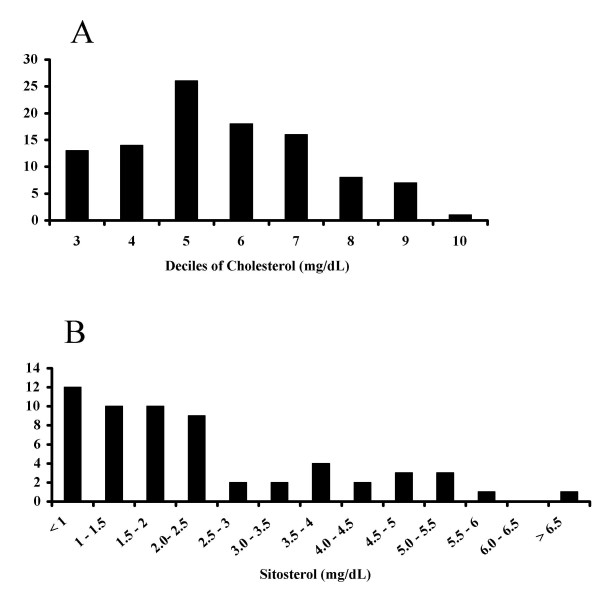
**Sterol distribution in F2 rats at 14 weeks age. **Blood for sterol analyses by GC was drawn at 14 weeks age (see Methods). The frequency distribution of cholesterol, in deciles, was almost Gaussian, with a slight right skew (panel A). However, plasma sitosterol, expressed in 0.5 mg/dl increments, was significantly right skewed (panel B).

To ensure there were no significant differences in growth curves of the F2 animals by Abcg5 genotype, body weights were measured serially and analyzed only after the genotypes had been determined at the end of the 14 weeks (Fig. 1). The genetic status at the *STSL *locus did not affect growth parameters. Blood from 14-week old F2 animals fed a chow diet was obtained after a 4 h fast and analyzed for cholesterol, as well as plant sterols, determined by GC analyses (see Methods). All sterol analyses were performed independent of the genotype analyses. Fig. 2 shows the distribution of cholesterol (Fig 2A) and sitosterol (Fig 2B) in these animals. The distribution of cholesterol was slightly skewed to the right, with a mean cholesterol value of 59.5 mg/dl. The distribution of sitosterol was significantly right-skewed and suggested a bi-modal pattern.

**Figure 3:**
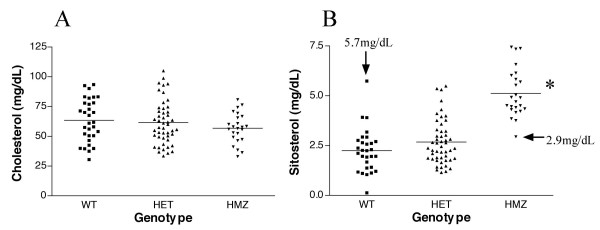
**Plasma sterols as a function of Abcg5 genotypes. **Cholesterol (panel A) and sitosterol (panel B) were segregated in all F2 animals by Abcg5 Gly583Cys. While there were no differences between cholesterol values between any of the genotypes, F2 rats who were homozygous for Cys583 (HMZ) showed significantly higher plasma sitosterol values (*, P < 0.05, mean 5.12 ± 0.25 mg/dl) compared to either heterozygous animals (HET, mean 2.68 ± 0.16 mg/dl) or wild-type (WT, mean 2.25 ± 0.20 mg/dl). These data were analyzed with the inclusion of two 'outliers' (arrows), one in the WT group and one in the HMZ group, where re-analyses to confirm these values were not possible (see Text).

All animals were genotyped for the Abcg5 G1757T mutation and grouped according to wild-type (WT), heterozygous (HET) or homozygous mutant (HMZ) and their plasma cholesterol (Fig. 3A) and sitosterol (Fig. 3B) values plotted as individual values. Two investigators, each blinded to each other's results performed the genotype and sterol analyses separately. There were no significant differences in plasma cholesterol values between any of the genotypes (Fig. 3A). All but two data points (highlighted in Fig. 3B) showed a robust segregation of homozygous inheritance of mutant alleles with elevated plant sterol levels. The two exceptions were a WT rat with an elevated plant sterol and a homozygous mutant rat that exhibited low levels of sitosterol. Unfortunately, the plant sterol levels were made available only after all of these animals had been sacrificed and it was not possible to re-sample the blood to re-confirm the plasma values, although archived DNA allowed us to confirm the genotypes. Thus an error in sample mis-identification could not be confirmed.

**Figure 4:**
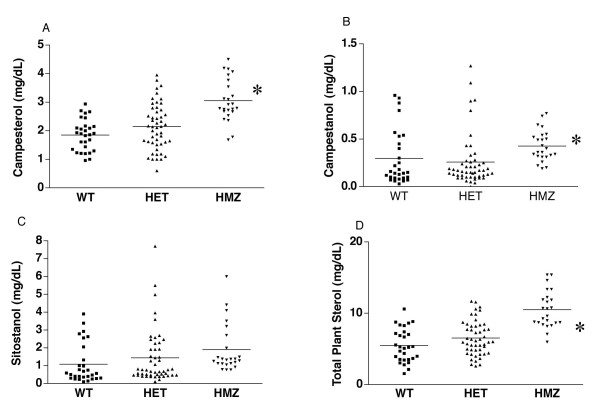
**Segregation analyses of other plant sterols by genotype. ** Segregation of campesterol (panel A), its metabolite, campestanol (panel B), the metabolite of sitosterol, sitostanol (panel C), or total plant sterols (panel D) are as shown. In all cases, except for sitostanol, the homozygous F2 rats had significantly elevated levels (* P < 0.05).

Plasma levels of campesterol, campestanol and total plant sterols were also significantly elevated in the HMZ animals compared to WT and HET animals (Fig. 4A, B and D), with non-significant increases in sitostanol (Fig. 4C). It is also interesting to note that a small number of the heterozygous animals had plasma sitosterol, campesterol and total plant sterol values as high as those from rats homozygous for the Abcg5 mutation. All of these measurements were obtained on animals fed a chow diet and a diet challenge to distinguish between these various genotypes was not performed.

### Intestinal absorption of lipid

**Figure 5:**
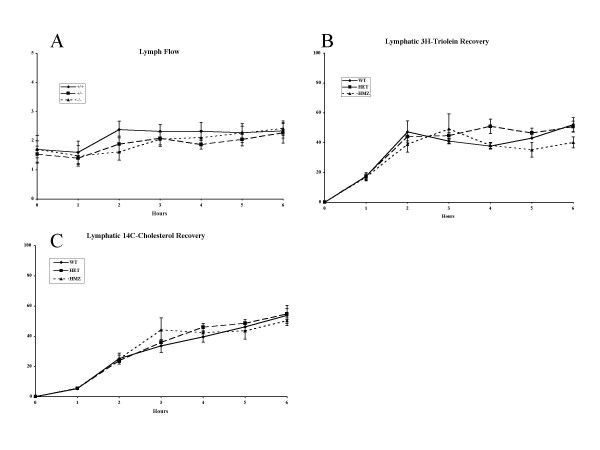
**Intestinal lipid absorption in F2 rats. **Male F2 rats had their lymph ducts cannulated as described (see Methods) and had lymph flow quantitiated to ensure adequate and stable flow (panel A). Labeled triolein (panel B) or cholesterol (panel C was administered via the duodenal tube, and the recovery of radio-isotope in the lymph quantitated with time. No differences in the appearance of label for either triolein (triglyceride absorption) or cholesterol was observed between WT, HET or HMZ F2 rats.

To identify possible mechanisms for the elevated plant sterols, we performed lymph duct cannulation in WT, HET and HMZ male rats and monitored absorption of cholesterol, and fat. Although we attempted lymph duct cannulation in 30 animals, we obtained data in only 3 WT, 4 HET and 4 HMZ animals due to either a high mortality or unacceptable lymph flow. Lymphatic flow rates in successfully cannulated rats are shown in Fig. 5A. There were no differences by genotype. Following a bolus delivery of radioactive cholesterol and triolein (see Methods), no differences were observed between WT, HET or HMZ rats for recovery of radioactivity in the lymph for either triglyceride or cholesterol (Fig. 5A and B), suggesting that these pathways were relatively normal. Lymphatic recovery for absorption of sitosterol was not performed.

### Biliary secretion of plasma cholesterol and plant sterols

**Figure 6:**
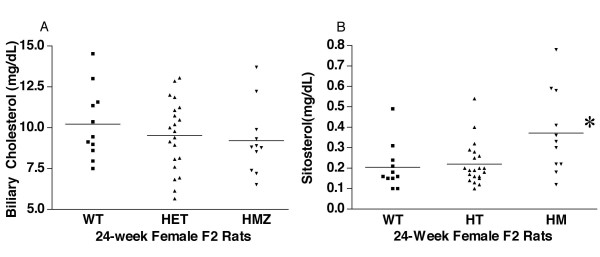
**Biliary cholesterol and sitosterol concentrations. **Female F2 rats, on a defined sitosterol diet (see text) had their bile ducts cannulated and bile collected under gravity and analyzed for cholesterol (panel A) or sitosterol (panel B). No differences in the cholesterol content of bile were noted between any of the genotypes. Note that the concentration of cholesterol is approximately 50-fold more than sitosterol. Interestingly, the sitosterol concentration in the F2 HMZ rats was increased significantly by ~ 50% relative to WT and HET rats (panel B).

To further elucidate the mechanism by which plant sterols were accumulating in the Abcg5 mutant animals, we collected and analyzed bile for sterols in 24-week old after they had been fed a defined diet containing 300 mg/kg of sitosterol (Fig. 6). There were no differences in the amounts of cholesterol present in the bile from any of the three genotypes (Fig 6A). However, significantly more sitosterol was present in the bile of mutant rats compared to either HET or WT rats (Fig 6B), suggesting that the excretory pathways are functional and not affected by this mutation. Thus the increased plasma plant sterol levels were reflected by the increased biliary secretion.

### Effect of Abcg5 mutation on blood pressure

**Figure 7:**
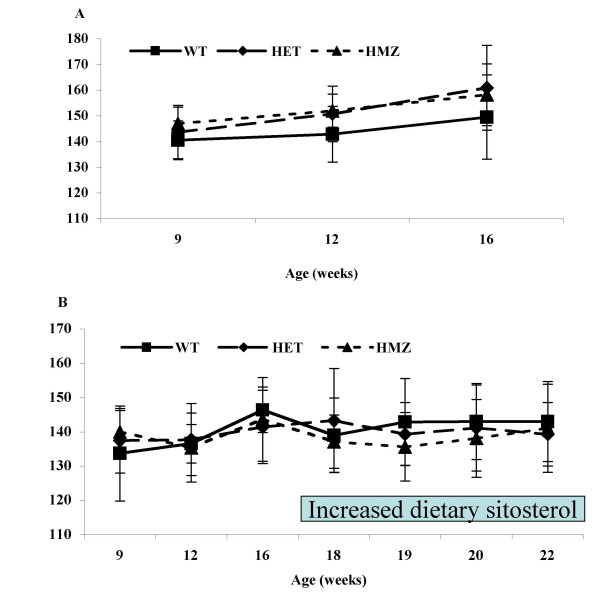
**Tail cuff blood pressure in male and female F2 rats with time. **Tail cuff measured blood pressures for male F2 rats (panel A) and female F2 rats (panel B) were monitored with time and analyzed by genotype (solid squares, WT; solid diamonds, HET and solid triangles, HMZ). No significant differences were noted between any of these genotypes. For comparison, the average blood pressures of rats at age 16 weeks for SD and SHR were 127 mm Hg and 197 mm Hg respectively. Additionally, female rats at 17 weeks were placed on a defined sitosterol diet and fed 1% NaCl drinking water (see bar, panel B). Neither significant increases in blood pressures, nor any differences between the genotypes were observed.

We used the tail-cuff measurement technique to monitor systolic blood pressure in these rats on a chow diet, with time. All blood pressure measurements were accumulated independent of the genotype results and only after all blood pressure measurements had been accumulated were the data analyzed by genotype (Fig. 7). In male (Fig 7A) or female (Fig 7B) mice on a chow diet, no differences up to 16 weeks of age were noted. For comparison, male SD rats had a mean systolic blood pressure of 128 mm Hg and the value for SHR was 197 mm Hg.

In a separate study, female mice were switched to a defined diet (containing 300 mg/kg plant) and had 1% NaCl added to the drinking water and blood pressures recorded for a further 5 weeks (Fig 7B, see bar). No differences in blood pressure were seen in any of the genotypes, nor was there a progressive increase in overall blood pressures recorded.

## Discussion

Sitosterolemia is a rare autosomal recessive inherited metabolic disorder of man that results in the accumulation on non-cholesterol sterols from the diet and is associated with premature coronary heart disease, hemolytic anemia and the formation of tendon and tuberous xanthomas [1]. The genetic defect(s) has now been mapped and identified. Mutations in one of two genes organized in tandem on human chromosome 2p21, ABCG5 or ABCG8, cause this defect [34]. As part of the literature survey, we observed that elevations of plant sterols had been reported in rats commonly used for hypertension and stroke research, WKY, SHR and SHRSP [19-25]. Interestingly, a number of studies had reported previously that alteration in diets not only increased the hemolysis associated with the presence of sitosterol in these rats, but had a dramatic effect on life-expectancy as well as led to an increase in hypertension [20,24]. Thus SHRSP rats fed canola or soy-bean oils supplemented with phytosterols led to a significantly shortened life-span relative to oils low in plant sterols [19]. Other investigators have reported the association of hypertension in these rat strains when fed diets supplemented with sitosterol, suggesting that hypertension may be modified by plant sterol accumulation [22].

Examination of SHR, SHRSP and WKY rats showed that these rats carried a homozygous missense in Abcg5, Gly583Cys, affecting a residue that shows conservation from Fugu to Man, despite the relative conservative change with respect to the amino acids involved [26,27]. At present, there are no control strains for WKY, SHR or SHRSP, since these strains have been maintained by brother-sister matings for >80 generations. Additionally, some of these strains have been maintained by different investigators for several generations raising the possibility that there may also be sub-strain variations between different colonies. Although these strains have been used extensively in genetic analyses of hypertension and a number of loci mapped for this trait, none of these loci map to the rat *STSL *locus on chromosome 6 [35]. In a literature search of genetic changes reported for these strains, we were able to compile at least 3 other loci that are mutated in the SHR strain (Cd36, Srebp-1 and Kat-2), suggesting the possibility that the missense 'mutation' identified in Abcg5 is not causative for sitosterolemia (which is also moderate in comparison to levels seen in patients with sitosterolemia who have levels frequently >15 mg/dl), but associated as a result of the in-breeding [29-31]. Additionally, a murine QTL study of plasma sitosterol levels showed that plant sterol levels were strongly influenced genetically, but mapping studies showed that this trait did not map to the murine *STSL *on chromosome 17 [33], raising the possibility that other loci/factors may be involved. Finally, in a study involving the GH and Norwegian Brown rat strains (neither of which are sitosterolemic), a QTL for hypertension was mapped to the region encompassing the rat STSL. However, we sequenced the rat *STSL *in both of these strains and did not identify any differences, suggesting that variations at the *STSL *in the rat may not be contributing to the hypertension [27]. We therefore undertook this study to test the hypothesis that the Gly583Cys was causative for sitosterolemia. We also measured blood pressure to test the hypothesis that this mutation was responsible for contributing to an elevation in blood pressure in these affected strains.

Segregation analyses in a large F2 population (SHR × SD) showed that homozygosity for Gly583Cys segregated with elevated plasma plant sterol levels in all but two animals. Of these latter animals, we could not verify that the samples had been inadvertently mis-labeled, as we could not repeat plasma analysis as the animals had been sacrificed before the plant sterol analyses were available. If we exclude these animals, all animals that are homozygous for the Gly583Cys are also sitosterolemic on a rodent chow diet, proving that this genetic change was responsible for the elevation in dietary non-cholesterol sterols.

We also tracked blood pressures, using tail cuff measurements, in all weaned animals for up to 4 months. No differences in blood pressures by genotype were observed in these F2 animals. In a sub-study, at ~ 4 months, female rats were switched to a more defined diet that had a higher level of plant sterols (300 mg/kg compared to ~ 130 mg/kg in most rodent chow preparations). This was done as normal rodent chow is known to lead to variable sterol absorption. Additionally, the rats were given drinking water supplemented with 1% NaCl to increase any manifestation of hypertension. Despite these interventions, rats homozygous for the Gly583Cys change did not show any significant increases in hypertension, relative to wild-type or heterozygous animals. Thus, in our study, we could not demonstrate an increase in blood pressures, contrary to other published data. There are several explanations for this discrepant finding. Firstly, our animals are F2 animals and since a number of loci are implicated in the hypertension phenotype, these should segregate independently of the *STSL *locus (except for one QTL for hypertension identified in the GH × BN experiment, but not reported for crosses involving SHR). Secondly, the levels of plant sterols we employed in our dietary studies are considerably less than used by others. In almost all of the studies reported where the exact amount of plant sterols were reported, these have been almost 10-fold or more higher than those used in our current study. Our justification for not using such large quantities is that it is unlikely such intakes reflect amounts that would be normally consumed and thus may not be 'physiological'. Thirdly, the blood pressure measurements were undertaken in animals fed plain drinking water and in all of the reported studies where hypertension was monitored, supplementation with a high salt intake (in water, diet or both) has been used. Finally, the majority of studies using higher plant sterols also used increase plant oils as well and the effect of these on altering the phenotype is not easy to discern.

We are aware of the limitations of the tail-cuff technique for blood pressure determinations and subtle, but significant differences, in blood pressure may have been masked by the use of this technique. Our data indicate that variations at the *STSL *locus may not be responsible for major effects in contributing to blood pressure. Any subtle effects will need to be explored in rat strains that are congenic for Gly583Cys on either the SHR or SD backgrounds.

Although the Gly583Cys change affects an amino acid residue that is conserved from Fugu to Man in Abcg5, this amino acid substitution is a relatively conservative change, with no both amino acids being aliphatic and small. To delineate possible mechanisms by which this genetic change altered dietary sterol trafficking, we compared intestinal absorption for cholesterol and triglyceride, using lymphatic duct cannulation studies. These showed that neither cholesterol absorption, nor triglyceride absorption (digestion, uptake, re-synthesis and secretion) were affected by this change. Note that patients with sitosterolemia, mutated in Abcg8, have been reported to manifest hyper-absorption of cholesterol, as well as plant sterols [34]. Unfortunately, sitosterol absorption was not determined at the same time, as these studies were performed by a core (PT, University of Cincinnati) not routinely using sitosterol. Biliary excretion of cholesterol and sitosterol was unaffected by the Gly583Cys alterations, although the homozygous mutant F2 rats showed increased amounts of plant sterols in their bile. Thus, although not definitive, this mutation may cause an increase in the intestinal absorption of non-cholesterol sterols, leading to increased plasma concentrations. Despite an increase in biliary secretion, a small net retention may result, accounting for the sitosterolemia. In this context, it should be pointed out that the levels of sitosterolemia observed in these rats are considerably lower than those seen in patients with sitosterolemia. Future experiments, using congenic mice and lymph duct cannulation will allow us to examine this hypothesis and delineate pathophysiology of this missense mutation.

## Conclusion

We report here segregation analyses that prove that the Gly583Cys alteration identified in WKY, SHR and SHRSP strains is causative for sitosterolemia, that this alteration may not be a significant contributor to the hypertension phenotype, and that it may be causing sitosterolemia by primarily increasing intestinal absorption on non-cholesterol sterols. Our study also highlights the need to develop lines congenic for the missense mutation on an SD background and the wild-type residue on the SHR background to allow for the study of Abcg5 function on its role in the diet-induced decrease in longevity in these hypertensive strains.

## Methods

### Animal breeding and husbandry

The Animal Care and Use Committee of Medical University of South Carolina approved all animal protocols. Inbred SHR (SHR/NCrlBR) rats and outbred CD^® ^(SD) IGS BR rats were purchased from Charles River Laboratories (CRL, Wilmington, MA). Animals were housed 2–3 rats/cage in a temperature-controlled room (22 ± 2°C) with a 12-hour light/dark cycle and fed Teklad Sterilized Rodent Diet (W) 8656 (Harlan, Madison, WI) with free access to water. To produce F1 progeny, (1 male and 1 female) SHR and (1 male and 1 female) SD were cross-mated and the F1 resultant progeny randomly mated (4 breeding pairs) to produce 103 F2 progeny. All F2 animals were fed standard rodent chow and had free access to water. At 17 weeks of age, female rats were fed a high sitosterol diet (sitosterol, 300 mg/kg and cholesterol, 150 mg/kg, Harlan Teklad diet) and their drinking water supplemented with 1% Sodium chloride for 6 weeks. Males were shipped to University of Cincinnati (PT lab) for lymph duct cannulation.

### Blood pressure and body weight measurements

Blood pressure (BP) was measured in the warmed, conscious, restrained state by using the photoelectric oscillometric tail-cuff method using a Natsume KN-210 machine (Natsume Seisakusho Co. Ltd., Japan). Animals were accustomized to the apparatus for 1 week, before recordings were collected. At each time point, at least 10 readings were obtained for each rat and the average of the readings taken. Body weights were recorded after each BP measurement. All data were accumulated independent of the genotype data.

### Genotyping

Blood samples were drawn at 14 weeks of age from all rats. In addition, blood, was obtained at 20 and 24 weeks from female rats; the plasma was saved at -70°C for sterol analysis and genomic DNA, isolated from WBC, used for genotyping by PCR and *Hae III *digestion as described previously [27].

### Bile collection

Under Isoflurane anesthesia, the bile duct was cannulated and bile collected for 15 minutes under gravity as described previously [16].

### Sterol analyses

Plasma and bile samples were processed according to the methods described by Heinemann [36]. Plasma total cholesterol levels were measured by using gas-liquid chromatography (GC), and plant sterol levels determined by GC-mass spectrometry with 5-cholestane and epicoprostanol as internal standards, respectively, as described previously. Total plant sterols included campestanol, campesterol, sitostanol and sitosterol. Campestanol and sitostanol are 5-dihydro derivatives of campesterol and sitosterol. Sterol analyses and genotype analyses were performed independently and only after completion of both were results linked.

### Intestinal absorption

The intestinal lymph duct was cannulated using a clear vinyl tubing (o.d., 0.8 mm) according to the method of Bollman et al. [37]. The second soft silicone tubing (o.d., 1.6 mm) was installed in the stomach through the fundus into the duodenum and secured with a purse-string suture. A purse-string suture (6-0 silk) and cyanoacrylate glue were used to secure the duodenal infusion tube. The gastroduodenal and lymph duct tubes were externalized through the right flank of the rat to allow easy access for infusion and collection. The skin, abdominal musculature and peritoneal layers were closed in a single layer (4-0 silk). The rats were kept in restraining cages at an ambient temperature of 30°C and allowed to recover from the anesthesia. The next morning, a lipid emulsion containing 6 μmol of ^3^H-triolein, 0.78 μmol of ^14^C-cholesterol, 0.87 μmol of egg phosphatidylcholine, and 5.7 μmol of sodium taurocholate (19 mM) in 1.5 ml of phosphate-buffered saline (pH 6.4) was infused at 1.5 ml/h for 6 h. Lymph was collected 1 h before the lipid infusion (fasting) and then hourly for 6 h. The volume of collected lymph was measured by weighting. Samples were counted for 10 min by a liquid scintillation spectrometer (Model TR 1900 tri-card; Packard).

### Statistical analysis

Statistical analysis was performed by program Prism 2.0. Comparisons of parameters among 3 groups were analyzed by one-way ANOVA with Newman-Keuls's post-test and t-test. All data are expressed as mean ± SD.

## Abbreviations

ABCG5, ATP binding cassette G5; SD. Sprague Dawley STSL, sitosterolemia locus; SHR, spontaneously hypertensive rat, SHRSP; SHR stroke-prone;

## Authors' contributions

The studies were designed by JC, HY and SBP and all experimental data obtained by JC, SZ, AB, WF and PT. Data analyses were performed by JC, SBP, AB, PT and GS. The paper was written by JC and SBP and edited by all the authors.
